# Understanding experiences of the Swedish health care system from the perspective of newly arrived refugees

**DOI:** 10.1186/s13104-018-3728-4

**Published:** 2018-08-28

**Authors:** Elisabeth Mangrio, Elisabeth Carlson, Slobodan Zdravkovic

**Affiliations:** 1 Department of Care Science, Faculty of Health and Society, Malmö University, Malmö, Sweden; 2 Malmö Institute for Studies of Migration, Diversity and Welfare (MIM), Malmö University, Malmö, Sweden

**Keywords:** Health care, Refugees, Health care access, Experiences

## Abstract

**Objective:**

Refugees seek medical advice for a variety of reasons. Previous research suggests that understanding the refugees’ experiences of and access to healthcare are important factors for improving their health as access to healthcare has been found to be a leading health indicator. Therefore, the aim of this study was to illuminate experiences of the Swedish health care system from the perspective of newly arrived refugees.

**Results:**

More than 70% of newly arrived refugees in the county of Scania were in need of health care during the last 3 months of 2015–2016. They did not seek care to the same extent as the general population. The main reasons were explained as too high costs, long waiting times and language difficulties. Some disclosed being denied access to health care for reasons, such as being denied care when seeking emergency room for stomach problems and being denied follow-up care for diabetes.

## Introduction

A migrant’s personal history is often marked by torture, trauma, loss of family members and the struggle of having to resettle in a new country [1]. Numerous refugees and asylum seekers undergo both physical and psychological stress in their country of origin, as well as during the transition to and arrival in the host country. This can increase the risk of them developing mental health problems [2]. Consequently, it is unsurprising that refugees, with their traumatic pasts, have higher rates of depression, anxiety and posttraumatic stress disorders than the general population [3, 4]. Newly arrived refugees (NAR) seek medical advice for a variety of reasons, the most common being musculoskeletal and pain issues, mental and social health issues, infectious diseases and longstanding undiagnosed conditions [1]. Post-migration resettlement stressors—such as social isolation, financial problems, employment difficulties, generational acculturation differences, culture shock and housing—can adversely affect refugees` mental and physical health [4–9].

The literature suggests that understanding the refugees’ experience of and access to healthcare are important factors for improving their health [10] as access to healthcare has been found to be a leading health indicator [11]. Even though refugees have a higher need for healthcare in comparison to others, they face substantial barriers when it comes to healthcare access [12] because of language difficulties and economic and cultural aspects [12, 13]. In the scoping review conducted by Mangrio and Sjögren Forss [14], only one Swedish study [15] has thus far focused on the experiences of refugees and their encounters with the health care system. As a result of Sweden receiving a substantial number of refugees the past 3 years and previous research indicating that refugees are suffering from poor health, there is an urgent need for investigating how the NAR perceive the Swedish health care system [16]. Therefore, the aim of this study was to illuminate experiences of the Swedish health care system from the perspective of NAR.

## Main text

### Material and method

The current study is based on analyses of two sets of data based on different research approaches. One quantitative and one qualitative study have been performed targeting health issues in NAR in the establishment settlement process in the region of Scania, Sweden. Inclusion criteria for the study were that the participants had to be newly arrived refugees with either temporary or permanent residence permits and participate in the mandatory public integration programme.

#### Quantitative data

The descriptive statistics were derived from a survey that included approximately 1700 migrants from 2015 to 2016, speaking Arabic or Dari, and participating in the mandatory public integration support programme. This programme includes compulsory civil and health information as part of the introduction plan for all NAR in the Scania region of Sweden. Data collection was conducted through a self-administered questionnaire that included questions about self-rated health, sleeping habits, level of education, well-being, accommodation type, social relations, work and access to health care. In total, 681 questionnaires were returned, resulting in a response rate of approximately 39.5%. The variables were summarized in terms of frequencies and percentages stratified by gender.

#### Qualitative data

The qualitative descriptive study was conducted during 2017 through individual interviews with 15 NAR families that had received their residence permits. Each family was considered as one individual interview. It focused on how they experienced their situation during the resettlement process in the county of Scania. The interviews covered topics such as how the family perceived their situation, health, struggles and hopes for the future. Shortly after the interviews, each was recorded and transcribed verbatim. The interviews averaged 36 min (minimum 17 and maximum 60 min). The transcribed interviews were analysed with content analysis by Burnard [17]. Open coding was performed in English and was used to create one category of text in the results, which were discussed between the authors. Adjustments were made until consensus was reached to ensure credibility.

### Results

First, the quantitative data will be presented by descriptive statistics, followed by the qualitative data. The study will focus on NAR in the resettlement process and their opinion and experience of the Swedish health care system since arrival in the country.

#### Quantitative data

The study included 681 NAR who answered the questionnaire, of whom 461 were male, 204 were female and 16 lacking the information on gender. 462 respondents were below 45 years of age whereas 307 between 18 and 34 years of age, the remaining 155 were between 35 and 44 years of age. Low educational level (9 years or less) was reported by 146 respondents (102 males and 44 females), 141 had a medium educational level (9–12 years) (94 males and 47 females) and 301 respondents had a high educational level (more than 12 years) (218 males and 83 females). A total number of 71 respondents (48 males and 23 females) were working apart from the establishment settlement process and 576 respondents (402 males and 174 females) were not. Different aspects of health care consumption are presented in Table 1. For each health care related question in Table 1, a Chi Square was conducted in order to check for sociodemographic differences by the variables gender, age and education. The last question in Table 1, regarding if the person has been in need of health care during the last 3 months but not sought it, is followed by a question about reasons for not seeking care, as illustrated in Table 2. The most common reasons were the following: *Too long waiting times,* with 46.4% for men and 57.5% for women; and *could not afford it,* with 39.1% for men and 28.8% for women. Another common reason was *Language difficulties,* with 33.6% for men and 26.7% for women. Under the answer “other reasons”, several different reasons for not seeking care were mentioned such as emotional issues, no confidence in doctors and difficulties with access.

**Table 1:** Health care in relation to sociodemographic factors

	Yes	No	P-value
a: Do you know where to turn when you need health care?			
Age, n (%)			0.83
18–34	260 (80.7)	62 (19.3)	
35–44	137 (80.1)	34 (19.9)	
45–54	80 (79.2)	21 (20.8)	
55–64	35 (85.4)	6 (14.6)	
65–80	3 (100)	0 (0)	
Gender, n (%)			0.70
Men	354 (80.6)	85 (19.4)	
Women	163 (81.9)	36 (18.1)	
Education, n (%)			0.04*
Low	116 (73.9)	41 (26.1)	
Medium	124 (82.7)	26 (17.3)	
High	269 (83.3)	54 (16.7)	
b: Do you think that you have access to health care?			
Age, n (%)			0.72
18–34	239 (76.4)	74 (23.6)	
35–44	113 (71.5)	45 (28.5)	
45–54	75 (73.5)	27 (26.5)	
55–64	28 (75.7)	9 (24.3)	
65–80	2 (100)	0 (0)	
Gender, n (%)			0.87
Men	318 (74.8)	107 (25.2)	
Women	141 (74.2)	49 (25.8)	
Education, n (%)			0.11
Low	103 (68.7)	47 (31.3)	
Medium	113 (79)	30 (21)	
High	234 (75.5)	76 (24.5)	
c: Have you sought any medical treatment?			
Age, n (%)			0.00*
18–34	150 (48.1)	162 (51.9)	
35–44	86 (53.8)	74 (46.3)	
45–54	63 (61.8)	39 (38.2)	
55–64	32 (86.5)	5 (13.5)	
65–80	2 (100)	0 (0)	
Gender, n (%)			0.46
Men	228 (53.4)	199 (46.6)	
Women	107 (56.6)	82 (43.4)	
Education, n (%)			0.53
Low	82 (54.3)	69 (45.7)	
Medium	84 (57.9)	61 (42.1)	
High	161 (52.3)	147 (47.7)	
d: Have you been in need of health care during the last 3 months but not sought care?			
Age, n (%)			0.22
18–34	96 (30.8)	216 (69.2)	
35–44	35 (22)	124 (78)	
45–54	23 (22.8)	78 (77.2)	
55–64	11 (28.9)	27 (71.1)	
65–80	1 (50)	1 (50)	
Gender, n (%)			0.05*
Men	125 (29,1)	304 (70.9)	
Women	40 (21,5)	146 (78.5)	
Education, n (%)			0.00*
Low	28 (18.7)	122 (81.3)	
Medium	33 (22.8)	112 (77.2)	
High	101 (32.8)	207 (67.2)	

* Significance level P < 0.05

**Table 2:**
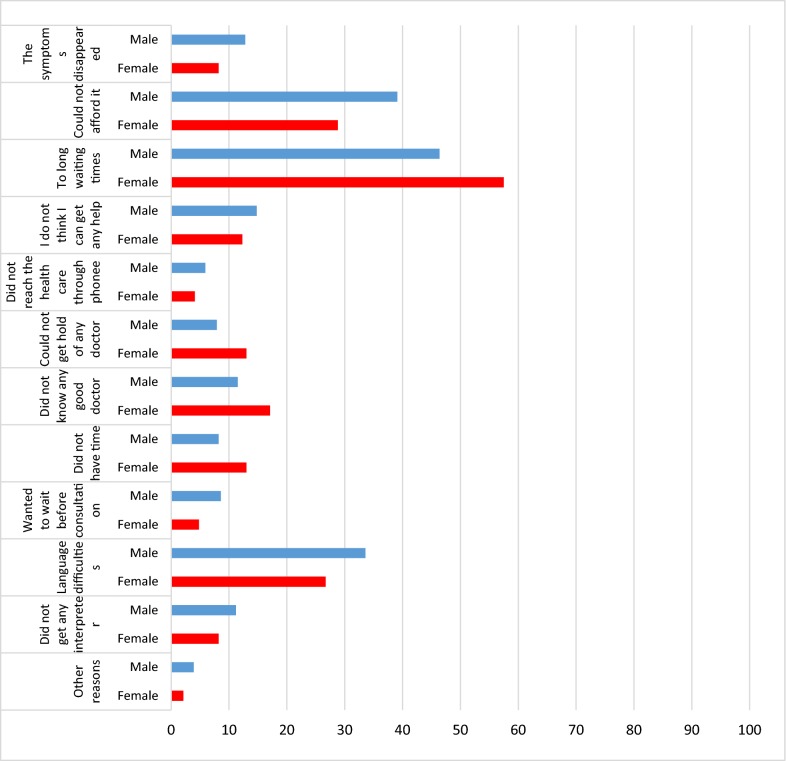
Reasons behind newly arrived refugees not seeking health care

#### Experience with the health care

The families described different experiences of the health care system. One family informed how their daughter received successful treatment at an orthopaedic clinic and how they were met by engaged doctors and nurses. However, other families were not as satisfied. Some related their struggles concerning making themselves understood at the primary health care centres, and many NAR were distressed and disclosed how they had expected better care elsewhere. One woman communicated:

*“I was at the health care centre for checking my coil and the problems with bleeding that I had. I got a doctor that was very unpleasant in her behaviour towards me. The treatments that I have got in Sweden are worse than what I have experienced before in my life”.* (Informant 11)

One woman concluded that in Syria patients with diabetes are better treated and taken proper care of, and are followed and monitored closely; however, she had not seen the same proper care in Sweden.

When asked about their health situation, several families answered that they had to wait long periods to get access to health care. Some referred to delays regarding registration at the health care centres, while others remarked on waiting for appointments after referral to different health departments. The families commented on the slow health care service in Sweden. One father remarked:

*“One time we went to the emergency department and had to wait for 24* *h before we got the help that we asked for. This is a problem here”*. (Informant 4)

Further, some of the families had experienced not getting access to needed health care on different occasions. One woman revealed that when her cousin sought medical for stomach pains help at a hospital the staff did not want to receive him. A month later, he was admitted to the hospital and had to have surgery because of the severity of the health issue. Another woman told about being denied care:

*“My asthma deteriorated during the asylum process. When I was asking for health care, they told me that I could not get it because I did not have my residence permit. They told me to come back as soon as I got it”.* (Informant 3)

One male informant was suffering from several health issues and had previously lived in the northern part of Sweden, where he was used to getting proper health care for his diabetes. However, having moved to a new town here in Scania, he encountered some difficulties receiving the care and follow ups he was used to. Another obstacle described by the migrants was the language barrier, which limited their possibilities for renewal of prescriptions through telephone consultations, for example.

### Discussion

NAR are facing challenges regarding encounters with the Swedish health care system. For example, they are met by health care professionals who display poor behaviour, they have to wait some time for referrals and appointments, and they are denied the health care they are in need of. This is in line with the results in the quantitative data, with nearly 71% of men and 78.5% of women responding to the item “have you been in need of health care during the last 3 months but not sought care”. This was clarified as a result of the following: not being able to afford health care, the long waiting times and language difficulties. Problems with long waiting times are not unique experiences: however, having to wait for care with limited knowledge of the language and understanding of the how the system works might add to feelings of stress. This implies that greater considerations need to be taken into account when securing health care adjusted to the migrants’ situation. Some of the families mentioned encounters with the health care system that they were not satisfied with. It might be of great importance that health care workers are prepared for handling culturally sensitive issues. Cultural awareness can be viewed as a critical factor and an essential component for providing relevant, effective and culturally responsive healthcare services to an increasingly diverse population worldwide [18]. For health care professionals to be culturally aware, genuine efforts are required to address cultural barriers through engagement in the community, as well as through working closely with members of the community in order to address the barriers [19]. Barriers—such as challenges with the encounters with health care personnel, having to wait for and being denied care—were all aspects that the informants in the present study seemed to have experienced. Therefore, there is an urgent need to see these barriers removed in order to help the NAR for improved encounters with the health care sector in Sweden. Information about the migrants’ experiences of the healthcare systems of their host countries is urgently needed to improve the quality of healthcare delivered and to provide opportunities for better access [14]. Consequently, this study is of importance in that it presents information about some of the newly arrived families’ experiences with the Swedish health care.

## Limitations

The strength of the current approach, combining two research methods, is quantifying the need and current knowledge in seeking health care, as well as illuminating a deeper understanding of NAR experiences of the Swedish system.

It is, however, important to note that the two studies were conducted during two different time-periods focusing on NAR. Although the two studies were conducted with NAR families as well as NAR individuals, we do not consider that it would affect the results as both genders are represented in the quantitative study. It is also important to note that both studies target the same outcome in the same phase of an early establishment process, as well as that the study participants were mainly from the same country of origin, i.e., Arabic-speaking.

The interviews were conducted mostly in Swedish, with Arabic interpreting, apart from two interviews that were conducted in Swedish and English. Interpreting increases a researcher’s potential study population, but poses challenges in terms of quality since interpretation could cause misunderstanding and miscommunication [20]. This needed to be considered when analysing the results of this study and optimising the outcome and trustworthiness of the interviews [20].

Another limitation regarding the quantitative study might be its response rate. However, an approximate dropout analysis has been performed by comparing the characteristics of the study participants with statistics from Sweden’s Division of Labour. The analysis suggested that people with higher levels of education are overrepresented in the present study [21]. It would have been considered beneficial if a greater population of migrants could have been investigated, but the overall aim and the study procedure was to include all NAR within the establishment process in the region of Scania over a period of 1 year. Performing the survey was only possible on a regional level, although a national level could have led to a significantly larger number of participants. More details regarding the survey are presented elsewhere [21].

## Abbreviations

EM: Elisabeth Mangrio
EC: Elisabeth Carlson
SZ: Slobodan Zdravkovic
AMIF: Asylum, Migration and Integration Fund
